# Understanding the microstructural evolution and mechanical properties of transparent Al-O-N and Al-Si-O-N films

**DOI:** 10.1080/14686996.2019.1666425

**Published:** 2019-09-25

**Authors:** Maria Fischer, Mathis Trant, Kerstin Thorwarth, Rowena Crockett, Jörg Patscheider, Hans Josef Hug

**Affiliations:** a Laboratory for Nanoscale Materials Science, Empa, Swiss Federal Laboratories for Materials Science and Technology, Dübendorf, Switzerland; b Solution Design, Evatec AG, Trübbach, Switzerland; c Department of Physics, University of Basel, Basel, Switzerland

**Keywords:** Aluminum oxynitride, Al-O-N, aluminum silicon oxynitride, Al-Si-O-N, coatings, thin films, transparent, hard, microstructure, reactive sputtering, 102 Porous / Nanoporous / Nanostructured materials, 103 Composites, 107 Glass and ceramic materials, 202 Dielectrics / Piezoelectrics / Insulators, 306 Thin film / Coatings, 503 TEM, STEM, SEM, 504 X-ray / Neutron diffraction and scattering, 505 Optical / Molecular spectroscopy

## Abstract

Optically transparent, colorless Al-O-N and Al-Si-O-N coatings with discretely varied O and Si contents were fabricated by reactive direct current magnetron sputtering (R-DCMS) from elemental Al and Si targets and O_2_ and N_2_ reactive gases. The Si/Al content was adjusted through the electrical power on the Si and Al targets, while the O/N content was controlled through the O_2_ flow piped to the substrate in addition to the N_2_ flow at the targets. The structure and morphology of the coatings were studied by X-ray diffraction (XRD) and transmission electron microscopy (TEM), while the elemental composition was obtained from Rutherford backscattering spectrometry (RBS) and heavy ion elastic recoil detection analysis (ERDA). The chemical states of the elements in the coatings were analyzed by X-ray photoelectron spectroscopy (XPS). Based on analytical results, a model describing the microstructural evolution of the Al-O-N and also previously studied Al-Si-N [1, 2, 3, 4] coatings with O and Si content, respectively, is established. The universality of the microstructural evolution of these coatings with the concentration of the added element is attributed to the extra valence electron (e^–^) that must be incorporated into the AlN wurtzite host lattice. In the case of Al-O-N, this additional valence charge arises from the e ^–^ acceptor O replacing N in the AlN wurtzite lattice, while the e ^–^ donor Si substituting Al fulfills that role in the Al-Si-N system. In view of future applications of ternary Al-O-N and quaternary Al-Si-O-N transparent protective coatings, their mechanical properties such as residual stress (*σ*), hardness (HD) and Young’s modulus (E) were obtained from the curvature of films deposited onto thin substrates and by nanoindentation, respectively. Moderate compressive stress levels between −0.2 and −0.5 GPa, which suppress crack formation and film-substrate delamination, could be obtained together with HD values around 25 GPa.

## Graphical abstract

**Figure:**
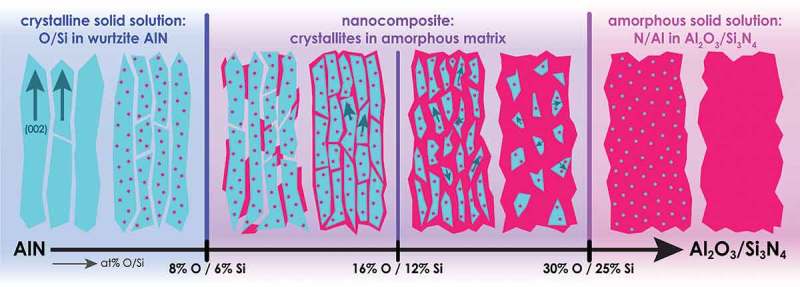

## 1. Introduction

Protective coatings are of critical importance for obtaining a performance enhancement of machinery and equipment or to protect the latter when used in harsh environments [5]. The properties and lifetime of a coating depend on its microstructure and physical and chemical properties. The chemical composition of the coating and the local chemical environment of its constituents must be controlled by the fabrication process and tuned to obtain optimized properties for specific applications. For example, the stress state of the coating must be optimized to prevent delamination and also to obtain closed boundaries between individual grains for a high inertness in corrosive environments.

Here, we are interested in protective coatings that are optically transparent, for example to protect optical devices or tools. A candidate material for transparent hard coatings is AlN, a group III nitride that forms as a polycrystalline wurtzite film when deposited by reactive direct current magnetron sputtering (R-DCMS). It has been observed that a microstructural film evolution can be induced by doping group IV and VI elements (Si, Ge, Sn and O) into AlN. Al-Ge-N [6], Al-Sn-N [7] and Al-Ge-O-N [8] are however not totally transparent but show colors ranging from yellow to red and brown, as the bandgap decreases from that of AlN with increasing contents of Ge and Sn. In our previous work [1–3], we have shown that Al-Si-N fabricated by R-DCMS is optically transparent and colorless for the complete range of Si contents tested.

For Si contents up to 6%, the films show a polycrystalline (002) oriented wurtzite structure with crystallites decreasing in size and a c-axis lattice parameter shrinking linearly for increasing Si concentration. We concluded that the lattice shrinking arises from Si incorporated in the wurtzite crystals in the form of a crystalline solid solution. For Si concentrations between 6 and 12%, the c lattice parameter remains constant. We concluded that an additional amorphous Si${\;}_{3}$N${\;}_{4}$ phase encapsulating the wurtzite grains, *i.e*. a nanocomposite, formed in this Si concentration range. A hardness (HD) maximum is obtained for about 10% of Si. For higher amounts of Si, a gradual loss of crystallinity is observed, and coatings with more than 25% Si were found to be fully X-ray amorphous [1–3].

In our previous work, the microstructure and properties of the films were modified by changing the content of the group IV element Si in the Al-Si-N films. Si with its low Pauling electronegativity $\chi_{Si}$= 1.8 replaces the electron (e${\;}^{-}$) donor Al with $\chi_{Al}$= 1.5 in the Al-N compound. In this study, we use O, an electronegative group VI element, to obtain a specific film microstructure and with it modified physical properties of the resulting Al-O-N films. Here, however, O with its high $\chi_{O}$= 3.5 replaces the e${\;}^{-}$ acceptor N with $\chi_{N}$= 3.0 [9] in wurtzite.

In addition to Al-O-N, we investigate quaternary Al-Si-O-N films fabricated by the simultaneous increase of the Si and O contents through the R-DCMS process. For both coating materials, Al-O-N and Al-Si-O-N, we report the changes in hardness (HD), elastic modulus (E) and residual stress (σ) that occur with changing microstructure.

## 2. Al-O-N thin film preparation and chemical analysis

Al-O-N coatings were fabricated by R-DCMS from metallic Al targets using O${\;}_{2}$ and N${\;}_{2}$ as reactive gases. The films were deposited onto Si(100) wafers and glass, and are 800–1200 nm thick, as measured by profilometry and confirmed by ellipsometry. The deposition parameters are summarized in Table 1 and detailed in the supplementary material (SM). Instabilities typically occurring in reactive sputter processes with O${\;}_{2}$ could be avoided by our modified sputter system setup described in [10].

**Table 1.** R-DCMS conditions used for the deposition of Al-O-N and Al-Si-O-N coatings.

R-DCMS deposition conditions	
chamber base pressure	$\leq 2\cdot 10^{-5}$ Pa
chamber volume	35 l
substrates	Si(100) and glass
substrate pretreatment	10 min RF at 75 V in Ar
substrate temperature	200${\;}^{\circ }$C
magnetron sputter geometry	dual unbalanced closed field
Al target power density	10 Wcm${\;}^{-1}$
Si target power density	0–5 Wcm${\;}^{-1}$
N${\;}_{2}$ flow to targets	12 sccm
O${\;}_{2}$ flow substrate	0–1.2 sccm
deposition pressure	0.2–0.4 Pa
target-substrate distance	12 cm
deposition time	3–5 h
resulting film thickness	800–1200 nm

Fully reacted, optically transparent Al-O-N films have a well-defined stoichiometry given by AlO${\;}_{1.5(1-z)}$N${\;}_{z}$ with z${\;}_{max}=1$ (AlN with 0% O) and z${\;}_{min}=0$ (Al${\;}_{2}$O${\;}_{3}$ with 60% O), as can be derived from the valences of the chemical elements of the films. The O content of the coatings deposited for this study was varied through the O${\;}_{2}$ flow in the deposition process. An O concentration range of 0.4–59.5% was achieved, as determined by Rutherford backscattering spectrometry (RBS) and elastic recoil detection analysis (ERDA). Coatings in the range of 8-16% were found to contain up to 3% H. Details for analytical techniques are given in the SM.

## 3. Analytical results obtained from Al-O-N films of different O content

### 3.1. Crystalline structure

AlN films deposited by R-DCMS for this study show only the wurtzite (002) peak and the higher order (004) peak in diffractograms from X-ray diffraction (XRD) θ-2θ scans symmetric to the AlN film surface (diffractogram profiles provided in the SM). This implies that AlN films are purely (002) textured in the growth direction (z). This is confirmed by a (002) pole figure (PF), which shows a single central pole for a sample inclination angle ψ = 0 (see SM).

θ-2θ scans in-plane, thus orthogonal to the sample surface and correspondingly to (002), show (100) and (110) signals. Both peaks have intensities that do not vary if the sample is rotated around its central z axis by φ. The crystallites hence have no preferred in-plane orientation. This is confirmed by (103) and (101) PFs, which show rims at ψ = 31.6${\;}^{\circ }$ and 61.6°, respectively, with constant intensities over the entire φ-circle. The AlN films thus have (002) fiber texture.

**Figure 1.**
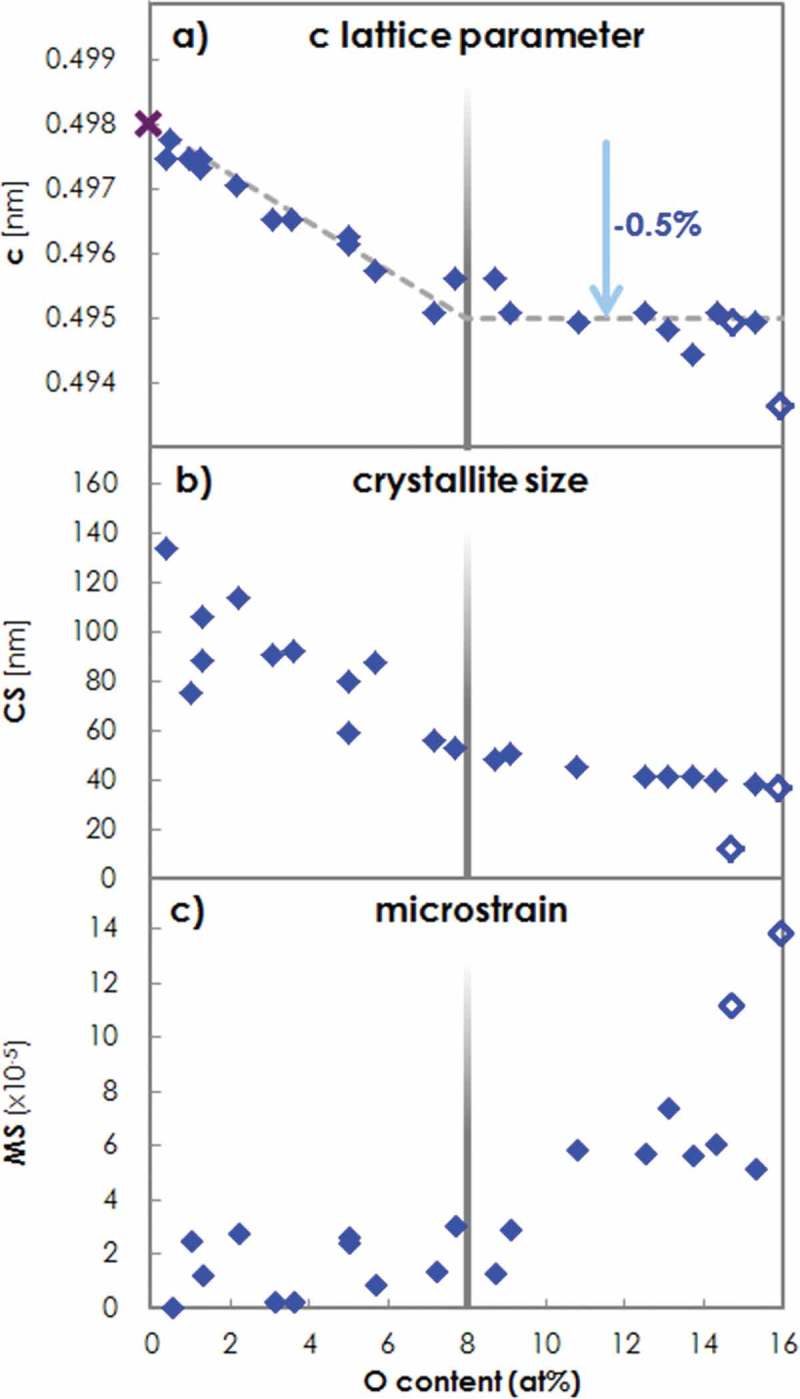
a) c-axis lattice parameter [nm] (found to be parallel to the (002) axis and the growth direction z) of wurtzite crystallites. The literature value for c in AlN [43] is marked by a purple cross. The dashed lines highlight the linear decrease of c observed between 0 and about 8% O, and that no further lattice shrinking occurs above 8% O. b) crystallite size (CS) [nm] and c) microstrain (MS) in Al-O-N plotted versus O content. XRD results of specific samples without uniaxial (002) texture with O concentrations below 16%, *i.e*. within the O content range typically leading to a (002) fiber texture, are shown by open symbols.

For Al-O-N films with an O concentration up to 8%, the (002) fiber texture of the film persists. The (002) peak shifts linearly to higher 2θ diffraction angles, broadens and decreases in intensity with increasing O concentration. The c-axis lattice spacing calculated from the (002) peak position, the crystallite size (CS) and the microstrain (MS) obtained from a line profile analysis (LPA) [11] using Scherrer’s equation for the CS, are plotted in Figure 1(a–c), respectively.

The wurtzite c-axis lattice parameter decreases by 0.5% from 0.4975 nm for AlN with residual O to 0.4950 nm for Al-O-N with 8% O content. Asymmetric θ-2θ scans reveal a lattice shrinkage also in the directions of (105), (104) and (103) for increased O contents and hence a contraction of the crystal unit cell. In previous work addressing the Al-Si-N system, the observed contraction of the wurtzite crystal lattice with increasing Si content was attributed to the generation of V(Al) vacancies in Al lattice sites. These V(Al) were proposed to form upon Si(Al) substitution as compensation for the additional valence e ^–^ in Si compared to the replaced Al [4]. As O also contains an additional e${\;}^{-}$ compared to N, we conclude that the O(N) substitution occurring in Al-O-N also leads to V(Al) and therefore to the observed wurtzite lattice shrinkage upon increasing O content.

Together with the crystal cell contraction, we observe a grain refinement (Figure 1(b)). The CS decreases from 140 to 60 nm in Al-O-N with an O concentration increased from about 2 to 8%. For films with a lower O concentration, scattering of the CS between 70 and 130 nm is observed, typical for materials exhibiting mosaicity. The MS (Figure 1(c)) remains constant around 2.10^–5^. (002) rocking curves (RCs) broaden with increasing O content (see SM), which indicates that the crystallites assume a progressive misorientation of the c-axis off the growth direction z.

No further shift of the (002) peak is observed for films with 8-16% O content. Consequently, the c lattice parameter remains constant. This behavior is similar to that found in the Al-Si-N system, where no further lattice shrinkage was observed for a Si content higher than 6%. We thus identify an O concentration of 8% as the solubility limit for O incorporated into the wurtzite lattice. Consequently, excess O of concentrations between 8 and 16% is incorporated in the form of a separate phase. As no additional diffraction peaks occur from this phase, and the films remain transparent, we conclude that the second phase consists of amorphous Al${\;}_{2}$O${\;}_{3}$ that encapsulates the AlN grains, hereby forming a nanocomposite. The CS was found to shrink slightly from 60 nm down to 40 nm (Figure 1(b)). The microstrain increases from about 2$\cdot 10^{-5}$ to about 6$\cdot 10^{-5}$ for an O concentration above 8%, indicating that the formation of the amorphous grain boundary phase exerts a pressure onto the Al-O-N grains.

At an O content approaching 16% O, wurtzite (100) and (101) peaks appear in addition to the major (002) peak in symmetric θ-2θ diffractograms (see SM). We conclude that at these high O concentrations the uniaxial (002) texture is lost, and crystallites with other orientations appear. The XRD results of two typical samples with such a mixed, yet highly preferred (002) orientation are plotted with open symbols in Figure 1. Their c lattice parameter and CS are lower, and their MS significantly higher compared to purely (002) textured samples.

For Al-O-N films with 16-20% O, the (002) peak develops an asymmetric shoulder towards lower 2θ, possibly arising from a weakly ordered grain boundary phase. For films with O contents of 20-30%, diffractograms show only small humps between 30 and 40${\;}^{\circ }$ in 2θ, which cannot be clearly attributed to a crystalline phase. We can thus conclude that at such high O contents of 16-30%, the coatings contain small, poorly aligned grains. The volume fraction of the crystalline phase decreases, while that of the amorphous tissue phase increases. Films with O contents beyond 30% are X-ray amorphous.

### 3.2. Cross-sectional film morphology

**Figure 2.**
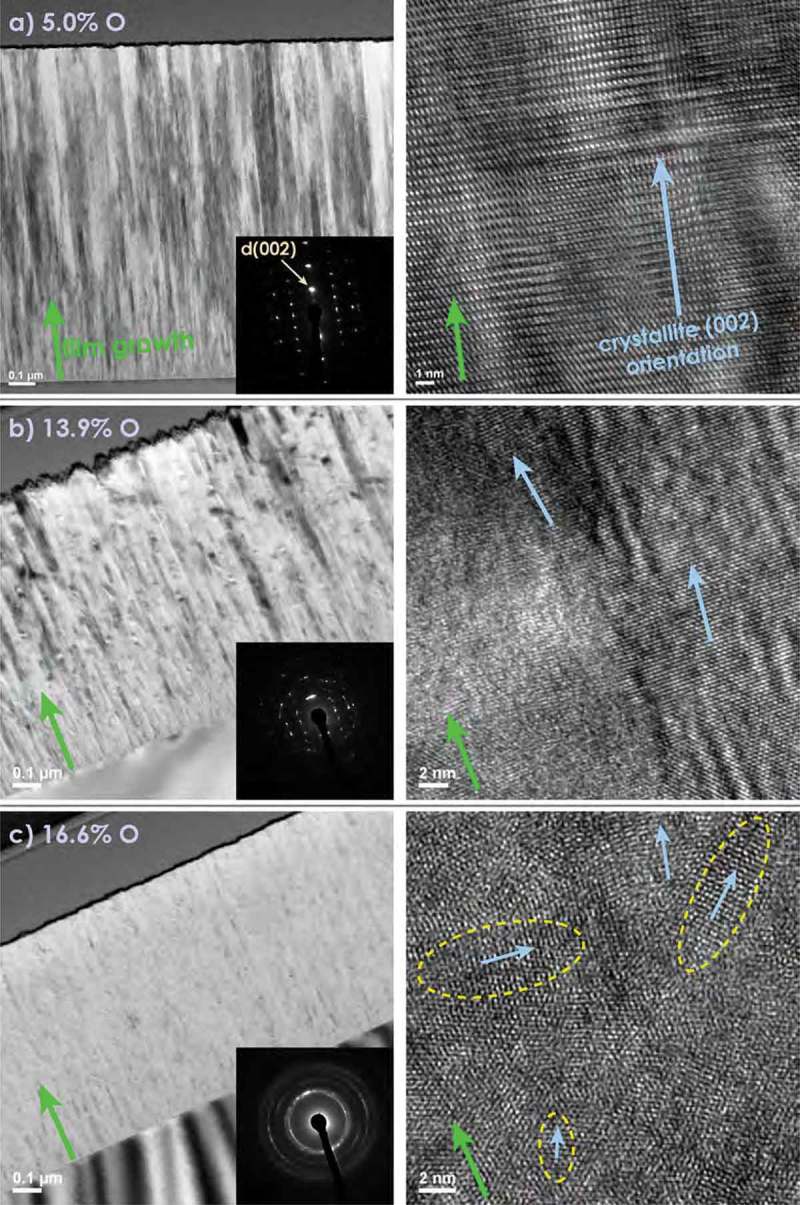
TEM images of cross sections of Al-O-N thin films with different O contents of a) 5.0% (top), b) 13.9% (middle) and c) 16.6% (bottom). On the left, a BF image and an ED pattern (small insert), and on the right, a HR image for each sample are shown. Film growth directions are indicated with green, crystallite (002) orientations with blue arrows. For the 16.6% O sample, nanometer-sized crystallites typically occurring for such an O content are highlighted by yellow, dashed ellipses.

Figure 2 shows cross-sectional transmission electron microscopy (TEM) bright field (BF) images (left), electron diffraction (ED) patterns (small insertions) and high resolution (HR) images (right) of samples with O contents of a) 5.0%, b) 13.9% and c) 16.6%.

The cross-sectional TEM of the film with 5% O in Figure 2(a) shows a columnar structure along the z-direction (green arrow). The ED pattern in Figure 2(a) contains sharp wurtzite diffraction spots. The HRTEM reveals large coherent crystalline regions. This confirms the (002) wurtzite fiber texture of the films revealed by our XRD analysis.

Figure 2(b) displays cross-sectional TEM data for a film containing 13.9% O. Again a fiber texture is apparent, but the ED pattern shows broadened spots, compatible with the decreasing alignment of the (002) axes of crystallites along the growth direction z.

The grain refinement (Figure 1(b)) and loss of (002) texture at higher O concentrations becomes apparent in the TEM results displayed in Figure 2(c), showing data obtained on a film with 16.6% O. Only faint columnar features are visible in the BF image. The ED pattern consists of rings with an increased brightness along the (002) direction. This indicates that (002) is still the preferred orientation that crystallites adopt in z, but that the pure (002) fiber texture observed in films with lower O contents is lost, and grains of random orientation exist. The latter is confirmed by the HRTEM data, where crystallites of 5–10 nm length with different (002) orientations are visible (encircled with dashed yellow lines in Figure 2(c)).

### 3.3. Chemical states

**Figure 3.**
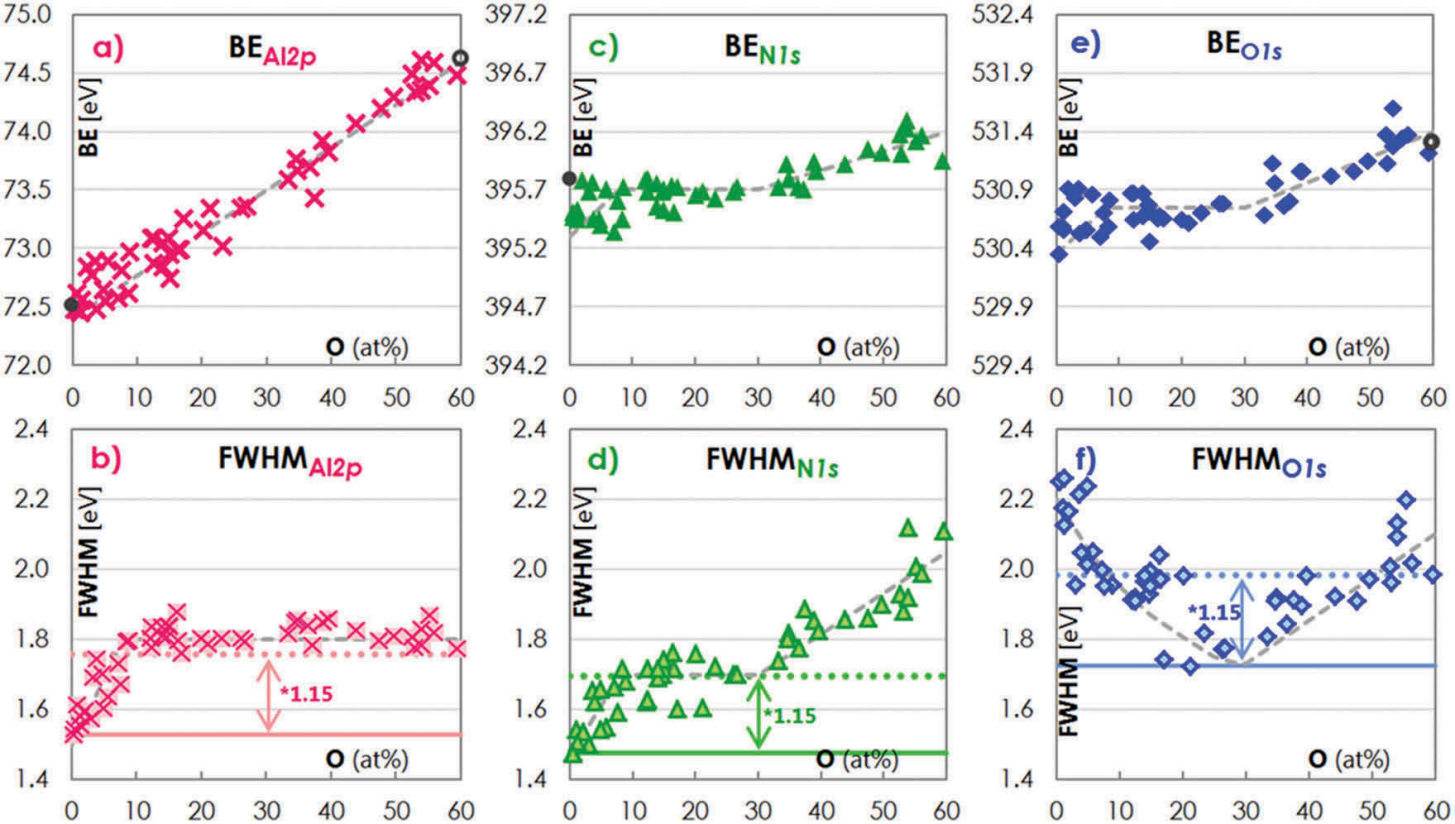
The top panels show the variation of binding energy (BE) with O content for the a) Al *2p*, c) N *1s* and e) O *1s* lines in XPS. The dashed gray lines highlight the evolution of the BEs on O content discussed in the main text. Literature values are marked with gray dots for AlN [13] and with gray rings for Al${\;}_{2}$O${\;}_{3}$ [14]. The bottom panels display the full widths at half maximum (FWHM) of the photo e${\;}^{-}$ lines b) Al *2p*, d) N *1s* and f) O *1s*. Again dashed gray lines are shown to highlight the observed dependence on the O content. Solid and dotted lines are added to the graphs highlighting the observation of a broadening by more than a factor 1.15 that has been previously indentified as a critical value for the occurence of disorder-induced line broadening [15–17].

The dependence of the chemical states of Al, N and O in Al-O-N films on their O content was analyzed by X-ray photoelectron spectroscopy (XPS), recording binding energy (BE) and full width at half maximum (FWHM) of photoelectron (photo e${\;}^{-}$) lines. Figure 3 shows the BEs (panels a), c), e)) and FWHMs (panels b), d), f)) of the photo e${\;}^{-}$ lines Al *2p*, N *1s* and O *1s* in Al-O-N coatings with increasing O content between 0.4 and 59.5%. This O content range corresponds to a variation of the material composition from close to AlN (0% O) over Al-O-N up to close to Al${\;}_{2}$O${\;}_{3}$ (60% O).

The BE${\;}_{Al2p}$ in Al-O-N is higher than in metallic Al, which has a BE${\;}_{Al2p}$ of 72.6 eV [12]. The reason for this is that Al in Al-O-N is oxidized as it acts as an e${\;}^{-}$ donor, because $\chi_{Al}$ of 1.5 is lower than $\chi_{O}$ of 3.5 and $\chi_{N}$ of 3.0 [9]. The BE_Al_*_2p_* increases linearly from 72.5 in AlN to 74.5 eV in Al${\;}_{2}$O${\;}_{3}$, *i.e*. with increasing O content up to 60% (see dashed gray line in Figure 3(a)). Concordant values for Al 2p in sputter deposited AlN of 72.5 eV [13] and Al${\;}_{2}$O${\;}_{3}$ of 74.6 eV [14] are reported (see gray circle and gray open circle in Figure 3(a)). The increase of BE${\;}_{Al2p}$ occurs as an increasing number of Al-N bonds are replaced by Al-O bonds. As O is more electronegative than N, Al-O bonds are more polar than Al-N bonds, thus the average oxidation level of Al and therefore BE${\;}_{Al2p}$ increase with increasing O content.

FWHM_A1_*_2p_* increases linearly from 1.5 to 1.8 eV with increasing O content from 0-8% (see dashed gray line in Figure 3(b)). In this O content range, XRD reveals that a solid solution forms, in which O populates the N-sites in wurtzite crystallites (section 3.1). While Al as e${\;}^{-}$ donor remains in cationic wurtzite lattice sites, incorporating O replaces N in anionic lattice sites, as both O and N are e${\;}^{-}$ acceptors. In wurtzite, cationic sites are coordinated to only anionic sites and vice versa. Al is thus directly connected to N and O. Hence, the variability of the Al oxidation states grows and the FWHM${\;}_{Al2p}$ broadens with increasing O content. Beyond the solubility limit of 8% O in wurtzite, FWHM${\;}_{Al2p}$ stays constant at 1.8 eV (see dashed gray line in Figure 3(b)). The reason for this is that from 8% O upwards, O is no longer incorporated into the AlN wurtzite lattice but an X-ray amorphous Al${\;}_{2}$O${\;}_{3}$ grain boundary phase, which increases in volume with increasing O content. In this phase, no higher oxidation level of Al than that in Al${\;}_{2}$O_3_ can form, thus the Al *2p* line does not broaden further. The FWHM${\;}_{Al2p}$ value for O contents beyond 8% is more than 1.15 times wider than the initial FWHM in AlN of 1.5 eV (see dashed gray line in Figure 3(b) and the dotted line highlighting the FWHM broadening by a factor of 1.15). We attribute this to significant disorder broadening due to a variation in the coordination around Al [15–17].

The BE${\;}_{N1s}$ in Al-O-N is lower than in molecular N${\;}_{2}$, which has a BE${\;}_{N1s}$ of 405.3 eV [18]. This is due to N acting as e${\;}^{-}$ acceptor, as $\chi_{N}$>$\chi_{Al}$. For an O content between 0 and 8%, Al-N bonds in the wurtzite lattice are gradually replaced by Al-O bonds. In addition, V(Al) vacancies appear to compensate for the extra e${\;}^{-}$ from O compared to N [4]. We thus expect the BE${\;}_{N1s}$ and FWHM${\;}_{N1s}$ to be modified by the presence of next-nearest neighboring O in the Al-O-N wurtzite unit cell and nearest neighboring V(Al). As $\chi_{O}$>$\chi_{N}$, we expect that O abducts e${\;}^{-}$ density from N with increasing O content, and that consequently the BE${\;}_{N1s}$ and the FWHM${\;}_{N1s}$ increase. The dependence of the BE${\;}_{N1s}$ on the O content (Figure 3(c)) is compatible with such a scenario, while the FWHM${\;}_{N1s}$ data clearly show a linear dependence on the O content (Figure 3(d)). The latter is because the V(Al) induce a variation and therefore an increased scattering of the BE${\;}_{N1s}$ (Figure 3(c)), thus leading to a significant broadening of the N *1s* line (see dashed gray line in Figure 3(d) and the dotted line highlighting the FWHM broadening by a factor of 1.15). For O contents between 8 and 30%, XRD reveals that no other crystalline phase than that of wurtzite containing 8% O is in the films, and that the CS in the latter decreases (section 3.1). It can therefore be proposed that an amorphous Al${\;}_{2}$O${\;}_{3}$ grain boundary phase of increasing thickness grows around the shrinking Al-O-N crystallites. This implies that the chemistry of the Al-O-N grains and thus the BE${\;}_{N1s}$ and FWHM${\;}_{N1s}$ do not change. For O contents beyond 30% the films are X-ray amorphous. As the atoms no longer sit on lattice sites in the amorphous Al-O-N phase, a direct O-N interaction exists. The latter further increases the BE${\;}_{N1s}$ as well as the FWHM${\;}_{N1s}$ (see dashed gray lines in panels c) and d) of Figure 3 for this O content range). In addition, a weak second N *1s* photo e${\;}^{-}$ line appears at a BE of 401.8–402.5 eV, compatible with the formation O-N bonds from partially oxidized N [19–23].

As with BE${\;}_{N1s}$, the BE${\;}_{O1s}$ of Al-O-N is lower than in molecular O${\;}_{2}$, which has a BE${\;}_{O1s}$ of 538.8 eV [18]. Also the dependence of the BE${\;}_{O1s}$ on the O content in Al-O-N is equal to that of the BE${\;}_{N1s}$ (compare Figure 3(c,e)). This is a result of both species being e${\;}^{-}$ acceptors. However, a pronounced difference between the dependence of the FWHM${\;}_{O1s}$ and that of the FWHM${\;}_{N1s}$ on the O content is evident (compare Figure 3(d,f)). The N *1s* line widens at high O concentration, while the O *1s* line widens at low O concentration. The reason is that at low concentration of each species, there is a wide variety of environments resulting in line broadening. The FWHM${\;}_{O1s}$ reduces to a minimum at 30% O. At this concentration, O predominantly exists in an amorphous Al${\;}_{2}$O${\;}_{3}$ grain boundary phase, while N is mainly present in the nanoscale Al-O-N grains. Above 30% O, both the FWHM${\;}_{O1s}$ and FWHM${\;}_{N1s}$ broaden due to the appearance of the additional N-O interactions in the amorphous Al-O-N phase.

## 4. Microstructural evolution model

**Figure 4.**
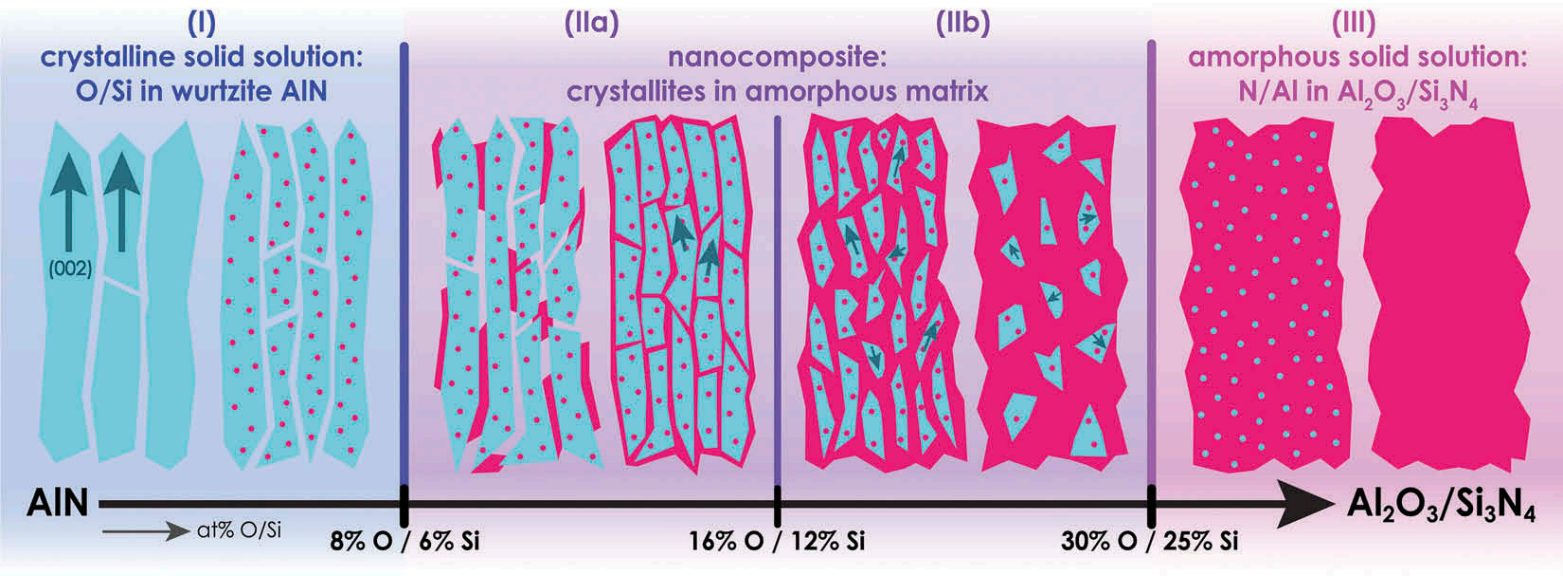
Microstructural evolution model of Al-O-N and Al-Si-N coatings with increasing O or Si content. Three regimes can be identified: Between 0 and 8% O (0 and 6% Si) a solid solution regime (I) exists. A shrinking of the c lattice parameter (Figure 1(a)) accompanied by a gradual refinement of the grains (Figure 1(b)) is observed. Between 8 and 16% O (6 and 12% Si) a nanocomposite sub-regime (IIa) occurs. The characteristics of the latter is a formation of an amorphous Al${\;}_{2}$O${\;}_{3}$ (Si${\;}_{3}$N${\;}_{4}$ for the case of Si added) grain boundary phase. In sub-regime (IIa) the (002) orientation of the crystallites remains along the growth axis z. This differentiates sub-regime (IIa) from (IIb). In the latter, the orientation of the crystallites along the 002 direction is gradually lost, as the O (Si) content is further increased from 16-30% O (12-25% Si). Above 30% O (25% Si), the coatings are X-ray amorphous.

We propose a model for the structural evolution of our Al-O-N films with increasing O content based on the experimental observations with XRD, TEM and XPS described above. The model, shown in Figure 4, is characterized by three regimes distinguished by O content.

At low O content of 0-8%, XRD and TEM reveal a wurtzite (002) fiber texture in the Al-O-N films. While the compositional analysis with ERDA and RBS shows that an increase in O leads to a decrease in N, XPS reveals that both O and N are electronically reduced (Figure 3(c,e)) as they act as e${\;}^{-}$ acceptors. It can therefore be concluded that O substitutes N in the wurtzite lattice. Upon these O(N) replacements, the wurtzite lattice shrinks linearly with increasing O content (Figure 1(a)) and the photo e${\;}^{-}$ lines Al *2p* and N *1s* exhibit a significant disorder broadening in the XPS spectra (Figure 3(b,d)). These observations can be attributed to vacancies in the Al lattice sites, as one V(Al) forms per each three O(N) in order to compensate the additional valence e${\;}^{-}$ that O has compared to the replaced N. Simultaneously to the crystalline changes in the wurtzite grains, XRD shows a grain refinement (Figure 1(b)) and a gradual loss of preferred orientation (XRD RCs) with increasing O concentration. This can be attributed to the e${\;}^{-}$ configuration of O, which mismatches the electronic structure of wurtzite and thus interrupts crystal growth. TEM images (Figure 2(a)) reveal crystalline domains in contact with each other. We can thus conclude that coatings with 0-8% O belong to a crystalline solid solution regime (I) (Figure 4), in which O (red dots) is integrated into wurtzite grains (green) exhibiting (002) fiber texture (green arrows).

At intermediate O concentrations of 8-16%, the c-axis lattice parameter of the crystal grains in the Al-O-N films remains constant (Figure 1(a)). This observation leads to the conclusion that the solubility limit of O in the wurtzite crystallites of the sputter-deposited Al-O-N coatings is reached at 8% O and that the structure of the wurtzite grains does not change with increasing O content. Consequently, above 8% O, the additional O must be contained in an Al${\;}_{2}$O${\;}_{3}$ grain boundary phase. This phase is not detected in XRD diffractograms and hence is amorphous. The microstrain increases in coatings with 8 to 16% O (Figure 1(c)), supporting the formation of a grain boundary phase that exerts a pressure onto the wurtzite grains. In XPS spectra, the photo e${\;}^{-}$ lines Al *2p* and N *1s* do not broaden further between 8 and 16% O (Figure 3(b,d)), an observation that also corroborates the existence of a second phase. XRD PFs reveal that the (002) fiber texture persists, while TEM images and XRD RCs show that the clear (002) alignment of the crystallites along the z-axis is gradually lost at higher O contents (Figure 2(b)). It can thus be concluded that Al-O-N films of 8-16% O are made up of a (002) fiber textured nanocomposite (IIa) (Figure 4), in which crystalline Al-O-N wurtzite grains with an increased (002) tilt (diverging green arrows) are progressively encapsulated in an amorphous Al${\;}_{2}$O${\;}_{3}$ matrix (red). At O contents of 16-30%, the XRD diffractograms show weak, broad peaks of (002) and further diffraction signals, and TEM reveals small crystallites oriented in arbitrary directions (Figure 2(c)). Coatings with more than 16% O thus exhibit a gradual loss of crystallinity and a loss of the fiber texture and therefore belong to the nanocomposite sub-regime without uniaxial texture (IIb) (Figure 4).

A third regime can be identified for O concentrations of 30-60%: XRD diffractograms of the Al-O-N films do not exhibit crystalline diffraction signals, signifying that the coatings are fully amorphous. XPS shows that the BE of all photo e${\;}^{-}$ lines as well as the FWHM of N *1s* and O *1s* increase, and that a second N *1s* component from oxidized N appears. These XPS results support the formation of an amorphous Al-O-N network in which bonds between all species exist. We conclude that coatings with more than 30% O consist of an amorphous solid solution (III) (Figure 4), in which progressively less N (green dots) is interspersed in an amorphous network consisting mainly of Al${\;}_{2}$O${\;}_{3}$ (red) up to the maximum of 60% O.

The microstructural evolution model of the Al-O-N system discussed here is reminiscent of the Al-Si-N discussed in earlier work [1–4], apart from a slightly lower solubility for Si in wurtzite and lower concentrations for the boundaries between regimes of the latter system. The concentration boundaries of the Al-Si-N system are also shown in Figure 4. It is noteworthy that in the Al-O-N system discussed here, the e${\;}^{-}$ acceptor N is replaced by the e${\;}^{-}$ acceptor O, while in the Al-Si-N system, the e${\;}^{-}$ donor Al is replaced by the e${\;}^{-}$ donor Si.

## 5. Material performance of Al-O-N coatings

The performance of protective hard transparent coatings in applications is governed by the residual film stress (σ), the hardness (HD) and Young’s modulus (E) and the refractive index (n). These parameters exhibit variations depending on the chemical composition of the coatings.

**Figure 5.**
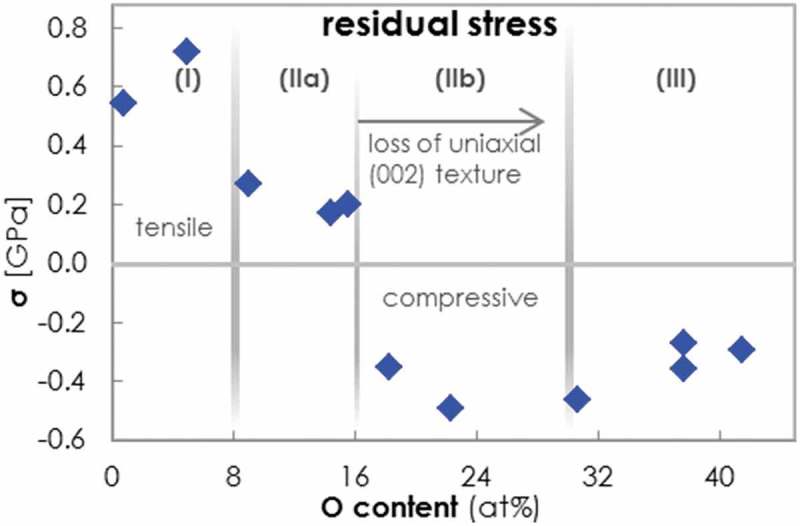
Evolution of the residual stress, σ, in Al-O-N coatings plotted versus the O content. σ was determined from films on extra-thin (30 μm Si(100)/145 μm glass) substrates, as these bend instead of leading to stress relaxation cracks in the coatings. The microstructural regimes are distinguished with gray boundary lines.

**Figure 6.**
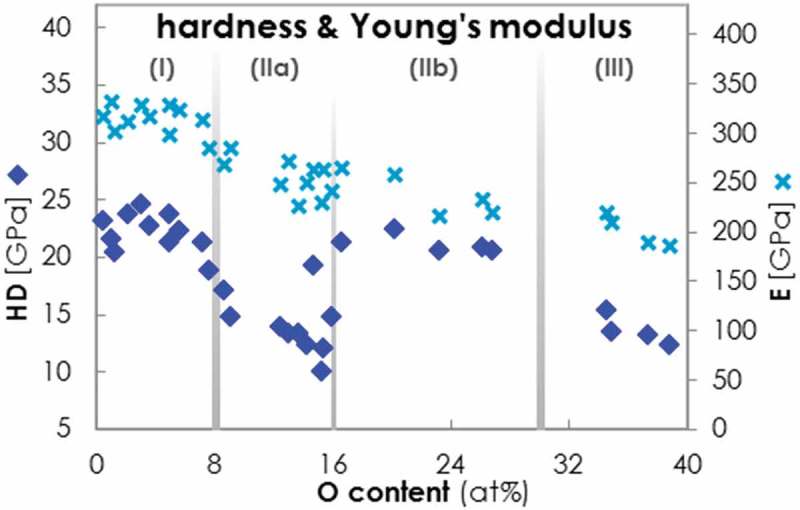
Evolutions of the hardness HD (blue rhombi, left axis) and Young’s modulus E (bright blue crosses, right axis) of Al-O-N coatings plotted versus O content. The microstructural regimes are distinguished with gray boundary lines.

The residual stress states σ of the coatings investigated in this study were determined from the curvature of extra-thin (30 μm Si(100)/145 μm glass) coated substrates. The curvature radii and film thicknesses were measured with a profilometer and fed into the Stoney equation (the modified form for Si(100), taking substrate anisotropy into account) [24]. The dependence of σ in Al-O-N coatings with increasing O content is shown in Figure 5. For all coatings, |σ| remains below 1 GPa. In the crystalline solid solution regime (I) containing 0-8% O, σ is tensile and reaches values of 0.6–0.8 GPa. We attribute the tensile stress to the presence of open grain boundaries and consequently to the occurrence of attractive intergranular forces [25,26]. We observed that this stress level is high enough to cause relaxation cracks on regular (not extra-thin) Si substrates. Cross sections at locations of film cracks, prepared and imaged with a gallium focused ion beam SEM (GaFIB-SEM), revealed that the cracks propagate through the whole film and end inside the Si wafer (see SM). This is typical for films that are stiffer than the substrate [27,28], as is the case for Al-O-N coatings of regime (I) on Si(100). E${\;}_{Al-O-N}$ is 300 GPa in regime (I) (see Figure 6 discussed below), while E${\;}_{Si}$ is 130 GPa in (100) and 169 GPa in (110) direction. The Si substrates were found to crack open along (100) planes, as the mismatch in E between Al-O-N and Si(100) is larger than between Al-O-N and Si(110), and as the surface energy for Si(100) is with 1.36 Jm${\;}^{-2}$ lower than that of Si(110) with 1.43 Jm${\;}^{-2}$ [29–31].

In the fiber textured nanocomposite sub-regime (IIa) at 8-16% O, σ remains tensile but is at a lower level of 0.2–0.3 GPa, a stress level that allows crack-free films on substrates with conventional thickness. The reduction in tensile strength occurs along with the encapsulation of the crystallites by the amorphous Al${\;}_{2}$O${\;}_{3}$ matrix in (IIa), which prevents attractive forces between the grains (Figure 4). It has been reported that there are additional mechanisms for the reduction of tensile or the increase of compressive stress attributed to a volume increase arising from the incorporation of O [25,32,33]. In the nanocomposite sub-regime (IIb) at 16-30% O, σ becomes compressive and reaches values down to −0.5 GPa. This happens together with the loss of the uniaxial (002) fiber texture.

In the amorphous solid solution regime (III), σ relaxes to moderate compressive stresses of around −0.3 GPa.

Irrespective of the stress level, all coatings adhere strongly to the substrates (Si(100) and glass) and no delamination has been observed. The evolution of the film stress of the A-O-N thin film system with the O content described here is similar to that of the Al-Si-N system with the Si content [1], supporting the universality of the structural evolution model for these systems.

HD and E of Al-O-N coatings measured by nanoindentation are plotted in Figure 6 against increasing O content. In regime (I), the coatings exhibit high HD values of 20–25 GPa; the same value range is found in literature for binary AlN films [1,34,35]. In sub-regime (IIa), the Al-O-N coatings undergo a pronounced dip in HD down to values around 10–15 GPa. This observation is somewhat surprising, because the Al-Si-N systems shows a HD maximum in this sub-regime of the structural evolution model. We attribute the observed reduction of the HD in the Al-O-N system to the inclusion of hydrogen (H) that was detected by He-ERD in concentrations up to 3% exclusively in this sub-regime. H terminates covalent interactions in a network with a single bond and can therefore reduce the cohesive strength of a material and with it the HD. We assume that incorporation of H stemming from adsorbed H${\;}_{2}$O is possible in (IIa), as the Al${\;}_{2}$O${\;}_{3}$ matrix forming in this sub-regime is hygroscopic [33,36] and under tensile stress (Figure 5). In comparison, the Si${\;}_{3}$N${\;}_{4}$ matrix in the Al-Si-N thin film system is less hygroscopic. Consequently, the hardness of the latter system is governed solely by the microstructural state of the film and not jeopardized by a H-induced weakening of the chemical bonds. In (IIb), the HD values of the Al-O-N system increase back up to a level of 20–25 GPa. We attribute this to the compressive stress in this sub-regime which may prohibit H incorporation. In fact, no H was detected for films in this sub-regime.

In regime (III), HD values decrease to around 15 GPa. Towards the oxidized side of Figure 6, Al-O-N coatings approach the value reported for sputter deposited amorphous Al${\;}_{2}$O${\;}_{3}$ of 11.5 GPa [37].

In contrast to the HD, the Young’s modulus, E, shows no dependence on the H incorporation in (IIa). E in Al-O-N coatings decreases continuously from 300 to 200 GPa as the O content increases from 0-40% over all three microstructural regimes.

**Figure 7.**
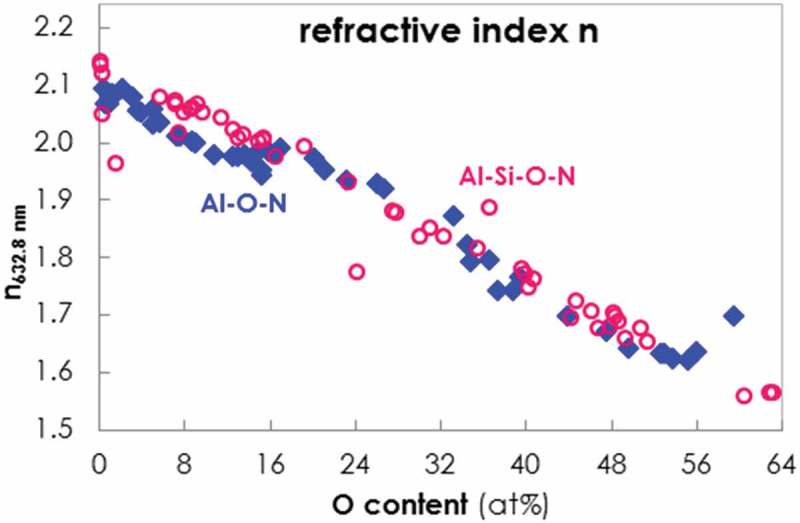
Evolution of the refractive index n${\;}_{632.8nm}$ of Al-O-N (blue rhombi) and Al-Si-O-N (open red dots) coatings plotted versus O content.

The optical properties of the coatings were determined by ellipsometry. While the extinction coefficient k was found to be zero for all films due to their transparency, the refractive index n${\;}_{632.8nm}$ of the Al-O-N films in dependence of the O content is shown in Figure 7 (blue rhombi). n${\;}_{632.8nm}$ decreases linearly from around 2.1 to 1.6 for an O content increasing from 0.4 to 59.5%.

AlN, Al${\;}_{2}$O${\;}_{3}$ and Si${\;}_{3}$N${\;}_{4}$ and also stoichiometric mixtures of these binary systems such as transparent Al-O-N, Al-Si-N and Al-Si-O-N are widely chemically and thermally inert. In addition, materials with an increased O content are less prone to post-fabricational oxidation [38–40]. In order to test the thermal stability of the fabricated Al-O-N coatings, high temperature *in situ* XRD (HT*is*XRD) was carried out for selected samples with O contents up to 16%. The tests showed a c-axis lattice parameter increase by 0.6% upon heating to 900${\;}^{\circ }$C due to the thermal expansion of the wurtzite lattice and full reversibility upon cooling. From this we conclude that the films are inert up to 900${\;}^{\circ }$C.

## 6. Quaternary Al-Si-O-N coatings

The XRD, TEM, and XPS results obtained on the Al-O-N system discussed above revealed a microstructural evolution of the system with increasing O content that is reminiscent of that observed for Al-Si-N system with increasing Si content described in earlier work [1–3] (Figure 4). We argued that the microstructural evolution in both systems is governed by the extra valence e${\;}^{-}$ that arises from e${\;}^{-}$ acceptor O replacing the N in the Al-O-N and from the e${\;}^{-}$ donor Si replacing the Al in the Al-Si-N systems. We thus expect that these mechanisms would also be present in the quaternary Al-Si-O-N films and that the boundaries between the regimes would be defined by the sum of the O and Si content. The data obtained on the quaternary system, however, reveals that this is not the case (see SM). We attribute this to the formation of Si-O bonds in the quaternary system, which is in competition with the replacement processes of the e${\;}^{-}$ donor Al by Si and of the e${\;}^{-}$ acceptor N by O. These latter processes drive the V(Al) formation and microstructural evolutions of the Al-Si-N and Al-O-N systems.

As in our earlier work [1–3], one Al and one Si target were used in the sputter deposition system to fabricate the quaternary Al-Si-O-N films at the conditions specified in Table 1. As for Al-O-N and Al-Si-N, the valences of the involved elements strictly define the stoichiometry for transparent Al-Si-O-N, which is given by AlSi${\;}_{x}$O${\;}_{y=1.5+2x-1.5z}$N${\;}_{z}$ with z${\;}_{max}=1+\frac{4}{3}$x for y=0. This can be derived from a mixture of the binary stoichiometries AlN, Al${\;}_{2}$O${\;}_{3}$, Si${\;}_{3}$N${\;}_{4}$ and SiO${\;}_{2}$. The Si content in the coatings fabricated for this study was varied through the power on the Si target and the O content through the O${\;}_{2}$ flow in the deposition process. A Si concentration range of 3.3–20.1% and an O concentration range of 0.1–63.2% were obtained, as measured by RBS/ERDA.

In the quaternary system, the residual stress σ shows no dependence on the chemical composition, such that all films exhibit moderately compressive stresses of around −0.5 GPa (see SM). This suppresses the formation of cracks in all Al-Si-O-N coatings independent of their chemical composition. Furthermore, Al-Si-O-N films show no significant H incorporation and thus no dip in the hardness HD. We observed that the HD of Al-Si-O-N depends only on the O, but not on the Si content. In Al-Si-O-N coatings of low O content, the HD is with 23–27 GPa slightly higher than that of Al-O-N, and decreases linearly to ∼8 GPa for Al-Si-O-N coatings containing around 65% O. Concomitantly with HD, the Young’s modulus E of Al-Si-O-N decreases linearly from 250 to 150 GPa with O increasing up to 65%. For high O contents, Al-Si-O-N shows similar HD and E values as glass consisting of amorphous SiO${\;}_{2}$, which has a HD of ∼8 GPa and an E of ∼75 GPa [41]. This suggests a structural similarity between Al-Si-O-N with low N content and glass, possibly arising from a large number of Si-O bonds.

**Figure 8.**
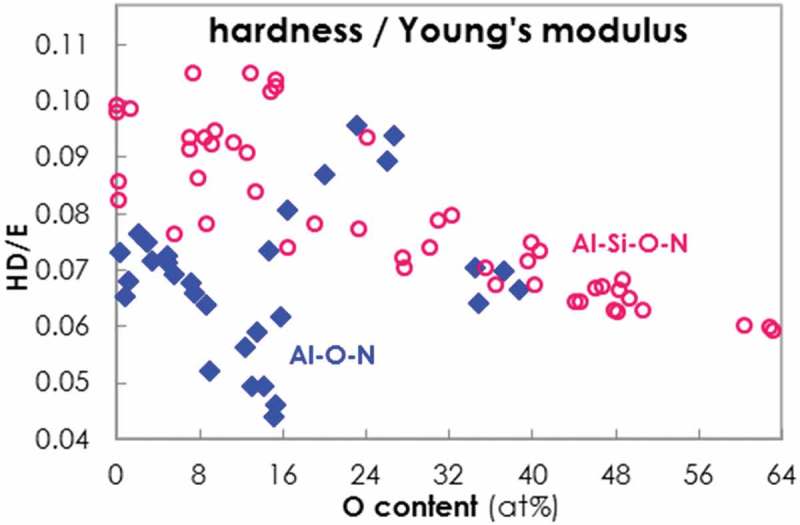
Evolution of HD/E (hardness divided by Young’s modulus) of Al-O-N (blue rhombi) and Al-Si-O-N (open red dots) coatings plotted versus O content.

The advantageous mechanical properties of protective coatings are often quantified by the HD/E coefficient (hardness divided by Young’s modulus) [42]. The higher the HD/E value, the more resilient, tough and fracture resistant a coating is. Figure 8 shows HD/E against O content for the Al-O-N and Al-Si-O-N systems. While HD/E is similar for Al-O-N and Al-Si-O-N of high O contents, Al-O-N shows a dip in HD up to 16% O. In this O content range, Al-Si-O-N clearly shows improved mechanical properties with an HD/E up to around 0.105. However, in the range of 16-30% O, Al-O-N has better HD/E values around 0.095.

Similarly to HD and E, the refractive index n${\;}_{632.8nm}$ of the Al-Si-O-N coatings, added in Figure 7 (open red dots), shows a dependence on only the O, but not the Si content. n${\;}_{632.8nm}$ of Al-Si-O-N exhibits the same linear decrease as that of Al-O-N, from around 2.1 to 1.6 for an O content increasing from 0.1 to 63.2%.

## 7. Conclusions

Transparent thin films of Al-O-N and Al-Si-O-N with different O and Si contents were fabricated by R-DCMS. The structure, morphology, hardness HD, Young’s modulus E and stress state σ of these coatings, the chemical states and bonding of the constituents were analyzed as a function of the O and Si content. We found that O addition to wurtzite induces the same microstructural evolution as Si addition, as both species lead to an $e^{-}$ excess. This commonality allows a general material evolution model to be proposed for both Al-O-N investigated in this study and Al-Si-N discussed in prior work [1–3]. In this evolution model, three regimes are distinguished by the O or the Si content: A crystalline solid solution regime (I), a nanocomposite regime (II) and an amorphous solid solution regime (III).

Microstructural tuning in thin films of the Al-Si-O-N system is therefore achievable by adjusting the chemical composition of the coatings through the sputter deposition conditions. In future work, R-DCMS may be used to fabricate coatings with vertical gradients. This could, for example, be achieved through a change of the sputter power on each of the targets or by changing the gas flow during deposition. The possibility of microstructural tailoring within gradient layers provides a powerful methodology for the design of coatings for specific applications. At the substrate-film interface, for example, the mechanical and optical properties of an Al-(Si-)(O-)N coating can be adapted to those of the substrate through HD, E and n. If the coating shall provide *e.g*. a diffusion barrier, a hard nanocomposite under moderate compressive stress and without open grain boundaries can be chosen for the bulk of the film. Towards the surface of the coating, the O content can be increased to obtain stability and inertness against post-depositional oxidation, or decreased to obtain a scratch-resistant coating with a high HD.
