# Supplementary material for: Understanding the microstructural evolution and mechanical properties of transparent Al-O-N and Al-Si-O-N films
Source: Sci Technol Adv Mater. 2019 Sep 25;20(1):1031–42. doi: 10.1080/14686996.2019.1666425 (PMC6844387; doi:10.1080/14686996.2019.1666425)
Supplement: Supplemental Material [file TSTA_A_1666425_SM1109.pdf]

Supplementary material

Experimental details

The **preparation** of the Al-(Si-)O-N thin films was performed in an AJA ATC 1500 F sputtering system (AJA International), using metallic Al and p-doped Si targets (Hauner Metallische Werkstoffe) and the reactive gases O_2 and N_2 (Messer Schweiz). The targets of 5 cm diameter were operated on unbalanced magnetrons in closed field configuration. The power on Al was 200 W for all experiments, while that on Si was varied from 20 to 100 W to obtain samples of different Si contents. N_2 at a constant flow of 12 sccm was introduced to the targets for all experiments, while O_2 at a flow varying from 0 to 1.2 sccm was fed to the substrate to obtain samples of different O contents. A custom-built setup, reported previously [10], was applied to avoid process instabilities due to the high reactivity of O_2, such that films with an arbitrary O/N content ratio could be deposited reproducibly. The chamber pressure during depositions amounted to 0.2-0.4 Pa. Polished Si(100) wafers (Silicon Materials (Si-Mat) and University Wafer) and conventional microscope glass slides (Menzel-Gläser) were used as substrates, and cleaned with an RF bias of 75 V for 10 min prior to depositions.

Rutherford backscattering spectrometry (RBS), **heavy ion elastic recoil detection analysis (ERDA)** and **helium elastic recoil detection (He-ERD)** measurements and evaluations were performed at the Laboratory of Ion Beam Physics (LIP) at the Swiss Federal Institute of Technology, Zürich (ETHZ). For RBS, samples were irradiated by 2/4/5 MeV ^{4}He, for ERDA by 13 MeV ^{127}I, and for He-ERD by 2 MeV ^{4}He. RBS was used to determine the compositional contents of Al and the major non-metal, *i.e.* either O or N. ERDA was then applied to refine the minor non-metal content via O/N ratio. He-ERD was used to probe the coatings for H. Further details on the compositional analysis of the samples is given in [10].

X-ray diffraction (XRD) was performed with three diffractometers. θ-2θ scans symmetric to the sample surface with the surface normal aligned to the scattering vector Q, rocking curves (RCs) and pole figures (PFs) were recorded on a Bruker D8 Discover with DaVinci design (Bruker AXS). In-plane θ-2θ and asymmetric θ-2θ scans were measured with a device of the same type, but equipped with a tube rotatable by 90°. High temperature *in situ* XRD (HT*is*XRD) was carried out on a PANalytical Xpert Pro (Malvern Panalytical) equipped with an Anton Paar XRK900 heating stage (Anton Paar).

All experiments were performed with a Cu anode operated at 40 kV and 40 mA. Cu Kβ was suppressed to 1% by a 0.012 mm thick Ni foil. For RCs, PFs, in-plane measurements, asymmetric XRD and HT*is*XRD, signals for combined Cu Kα_{1+2} (0.15418 nm) were evaluated. For symmetric θ-2θ, Cu K α_1 (0.154056 nm) components were extracted. All measurements apart from PFs were recorded with the beam in line-focus and parallelized by a primary Göbel mirror, while PFs were recorded in point-focus

Information Classification: General

and Bragg-Brentano geometry. All measurements apart from RCs were performed with the detector in 1D mode, while RCs were recorded in 0D mode. Data were analyzed with proprietary software and with Matlab (The MathWorks, Inc.).

Symmetric θ-2θ scans were recorded to obtain information about crystal planes parallel to the sample surface and thus orthogonal to the film growth direction (z). The obtained line profiles (LPs) are shown in figs. 9 and 10 and were analyzed via line profile analysis (LPA) described in the book of Birkholz [11], pages 85-101. A pseudo-Voigt (pV) function was used for the fitting of each wurtzite (002) peak, separating Cu Kα_1 and Kα_2. The peak position of the Kα_1 component was converted into the c-axis lattice spacing of the unit cell. The peak profile of the Kα_1 component was deconvoluted into its Cauchy and Gauss contributions. The Cauchy contribution to the peak broadening, assessed through the integral breadth, yielded the crystallite size (CS) via Scherrer's equation and the Gauss contribution the microstrain (MS) in the measured film. The instrumental broadening was accounted for via Si(400) peak obtained from the wafer substrate. RCs on the (002) peaks were analyzed for their FWHM, shown in fig. 11, as a measure for the angular distribution of crystallite tilts away from z. For the RCs, 2θ was fixed at the measured Cu Kα_{1+2} maximum of the wurtzite (002) peaks, and ω was rocked from 0°-2θ.

Pole figures (PFs) of wurtzite (002), (103) and (101), shown in fig. 12, were taken to explore the 3D texture of coatings. The “thinned” PF mesh function of the software was used with a delta of 2°, giving 5000 measurement spots distributed on 40 ϕ circles equidistantly spaced on the 0-80° ψ radius. For the (002) PF of a film, the 2θ value effectively measured in the symmetric θ-2θ scan of the respective film was used. For (103) and (101) PFs, the literature 2θ values [43] of 37.98° and 66.12°, respectively, were used.

In-plane θ-2θ scans were recorded to gain information about crystal planes perpendicular to the sample surface. The X-ray source was rotated by 90° in order to improve the signal intensity diffracted by the thin films. The line-shaped beam could thus be diverted into the film via Snellius angle and traverse the sample in parallel to the film surface, while probing it with its full length. Asymmetric θ-2θ scans on wurtzite (105), (104) and (103) yielded information about crystal plane dimensions aslope to the sample surface. (105) was measured at the sample inclination angle ψ=20.28°, (104) at 24.79° and (103) at 31.63°.

During high temperature *in situ* XRD (HT*is*XRD) up to 900°C, the heating stage was constantly flushed by Ar. After each heating step of 50°C, the sample was equilibrated at the corresponding temperature for 1 h and measured with a symmetric θ-2θ scan. A further scan was taken after re-cooling of the sample. Height variations due to thermal expansion of the sample holder were corrected by a stage mover.

Transmission electron microscopy (TEM) to image the cross-sectional structure of Al-O-N coatings was carried out on a JEM-2200FS (Jeol Germany). Films on Si(100) were broken, shaped into a wedge by mechanical tripod polishing, and finally ion-milled to e^- transparency. The wedge was contacted to a Cu ring with an inner diameter of 2 mm and then imaged in regions of 20-30 nm thickness. Bright field (BF) and dark field (DF) images, electron diffraction (ED) patterns and high resolution (HR) images were recorded.

Information Classification: General

X-ray photoelectron spectroscopy (XPS) was performed on a PHI Quantum 2000 Scanning ESCA Microprobe System (Physical Electronics (PHI)). Monochromatic Al Kα radiation of 1486.6 eV was used with a beam diameter of 150.0 μm. The base pressure in the measurement chamber was below $8 \cdot 10^{-9}$ mbar. The energy scale of the spectrometer was calibrated with the binding energy (BE) of the photoelectron (photo e^-) lines Cu $2p_{3/2}$, Ag $3d_{5/2}$ and Au $4f_{7/2}$ at 932.62, 368.21 and 83.96 eV, respectively, to within $\pm$ 0.1 eV [ISO 15472; 2010-05]. The FWHM of Au $4f_{7/2}$ amounted to 0.72, 0.93 and 1.57 eV for pass energies of 5.85, 29.35 and 117.4 eV, respectively.

Prior to measurements, the films were sputter-cleaned by an Ar ion bombardment at 1 kV during 600 s to remove the oxide natively forming upon atmosphere exposure and eventual carbonaceous contaminations. During measurements, the insulating samples were charge neutralized by low energy Ar ions and e^-. To check for and, if necessary, correct for a potential charge buildup despite charge neutralization, an established Au referencing method [13] was applied. For this, the molybdenum (Mo) masks used to clamp the samples during XPS measurements had been coated with 100 nm Au beforehand, so that a small amount of Au was re-deposited on the sample surface during sputter cleaning for oxide removal.

Spectra of the photo e^- lines Al *2p*, Si *2p*, O *1s* and N *1s* were measured at a pass energy of 29.35 eV and with 0.125 eV increments. For each peak, the BE and the FWHM were extracted. Spectral processing, shown exemplarily for an Al-Si-O-N film in fig. 13, was carried out in the software CasaXPS version 2.3.17PR1.1 (Casa Software Ltd.). Relative sensitivity factors were adopted from the system manufacturer PHI. Shirley backgrounds were applied. All peaks were symmetric and could be fitted with a single Gaussian-Lorentzian curve (GL(Gaussian percentage)). GL(30) for Al *2p* and Si *2p*, GL(40) for O *1s* and GL(60) for N *1s* were found to suit best. For N *1s*, the second peak appearing at high BE for some samples was best fitted with GL(20).

Ellipsometry to assess the refractive index (n) and extinction coefficient (k) of coatings was measured on a spectroscopic ellipsometer M-2000 VI (J.A. Woollam). The linearly polarized beam with wavelengths in the range of 400-1700 nm was set to irradiate coatings on Si(100) wafers under three angles of 50, 60 and 70. Measurements were recorded in reflection. The spectral dependence of the amplitude ratio *tan*(Ψ) and phase shift Δ in the elliptically polarized beam was simulated with a Cauchy model to reproduce the measurement results, as shown in fig. 14. For all Al-(Si-)O-N samples, the simulation was in alignment with measurement curves for k=0 in the wavelength range 400-1700 nm. The refractive index n at the red wavelength 632.8 nm ($n_{632.8nm}$), the emission line of a 85% helium and 15% neon gas laser, was extracted.

To analyze **biaxial residual stress (σ)** in Al-(Si-)O-N films, depositions onto extra-thin substrates were carried out. Either round Si(100) wafers of 30 μm thickness and 5 mm diameter (University Wafer) or microscope cover glasses of 145 μm thickness and 30 mm diameter (Menzel-Gläser) were used. These bend under the force of residual film stress and do not give way to stress relaxation through cracks, which would falsify obtained σ results. The radius of curvature of the bent substrates and the thickness of films were measured by a Bruker DektakXT contact profilometer (Bruker Germany) and by a Leica DCM8 optical profilometer (Leica Microsystems (Schweiz) AG) and applied in the Stoney equation [24], in a modified version for Si(100) substrates, to yield the residual film stress σ.

Information Classification: General

The thermally induced σ [[44]$^{formula12-20}$] upon sample heating to 200 °C during the depositions and subsequent cooling amounted to approximately -200 MPa. Tensile stress imposes a bending of the substrate rim upwards in film direction and shapes it into a concave lens, while compressive stress deforms the substrate convexly. By definition, tensile stress has a positive and compressive stress a negative sign.

On substrates of conventional thickness, stress-induced cracks were observed in crystalline Al-O-N films of regime (I) (see section 5). Cross sections at locations of such stress-induced film cracks were prepared and imaged with a Gallium Focused Ion Beam SEM (GaFIB-SEM) FEI Helios NanoLab G3UC (FEI Company). A typical Ga-FIB-SEM image, showing a crack propagating along line of sight, is given in fig. 15.

Nanoindentation for the hardness (HD) and Young's modulus (E) of the coatings was performed on a Hysitron UbI 1 (Hysitron, Inc.) equipped with a diamond Berkovich tip (SYNTON-MDP LTD.). Tip area calibrations on fused silica were regularly performed in between measurement sequences of 10-15 samples. Indentation load forces were chosen so that the resulting indent depths did not exceed 10% of the coating thickness in order to avoid an influence of the substrate on the measurement. 3 mN and/or 2 mN were found to be adequate and were used in quadratic 3x3 indentation matrices, in which the 9 single indents were spaced by 40 μm. Depending on the thickness (800-1200 nm) and the HD (8-28 GPa) of the coatings, either both matrices or only the 2 mN matrix were taken. Typical indentation depths were in the range of 60-100 nm. For samples, for which the 3 mN matrix did not lead to too deep indents, no systematic difference between the 3 mN and the 2 mN matrix was found. The results were evaluated according to Oliver and Pharr [45].

Experimental data obtained for Al-Si-O-N

The microstructural evolution of quaternary Al-Si-O-N with the total Si and O content is found to be far more complex than that of the ternary systems. We attribute this to the formation of Si-O bonds which compete with the roles Si and O play for the microstructural evolution of the corresponding ternary coatings. No well-defined regime boundaries could be established for the quaternary system (see section section 6). This is shown by means of XRD data in fig. 16.

Four types of diffractograms are distinguished, and the compositions of the films yielding the line profiles representative for each type are given. Type I shows a dominant wurtzite (002) peak, which is right-shifted compared to that of pure AlN [43] This indicates that a Al-Si-O-N film contains crystallites with a shrunken c-axis wurtzite lattice parameter. Weak wurtzite (100) and (101) peaks also appear. Type (II) shows the right-shifted (002) peak with a tail extending towards lower 2θ values. This tail possibly arises from a partially ordered grain boundary phase, or from a (100) component that is right-shifted due to a shrunken a-axis lattice parameter. In type III, the intensity of this tail component is increased. In type IV, a broad peak appears between 34 and 35° in 2θ, while the intensity of the right-shifted (002) peak decreases.

Information Classification: General

There is a trend for films with higher (O+Si) content to yield diffractograms with a higher type number, but an unambiguous assignment of distinct chemical composition regimes to diffractogram types is not feasible. The film with the highest (Si+O) content showing crystalline diffraction signals contains 5.6% O and 17.4% Si and thus a total of 23%. Films with higher (Si+O) concentrations are X-ray amorphous.

As discussed in section 6, the residual stress σ of the quaternary Al-Si-O-N coatings, shown in fig. 17, remains moderately compressive around -0.5 GPa with varying chemical composition.

Figures for the supplementary material

Figure 9. Normalized diffractogram line profiles from symmetric θ-2θ scans recorded with Cu Kα on Al-O-N thin films containing 0.4-14.3% O. The relevant 2θ range 35.6-36.8°, containing all signals arising from the coatings, is shown. The reference position of the wurtzite Cu Kα_1 peak [43] at 36.07 $^\circ$ for (002) is shown in black. Fitted Cu Kα_1 components of the coatings, fed into LPA, are added with thinner lines.

Figure 10. Normalized diffractogram line profiles from symmetric θ-2θ scans recorded with Cu Kα on Al-O-N thin films containing 14.7-34.6% O. The relevant 2θ range 30-40°, containing all signals arising from the coatings, is shown. Reference positions of wurtzite Cu Kα_1 peaks [43] at 33.26 for (100), 36.07° for (002) and 37.98 for (101) are shown in black and gray. Fitted Cu Kα_1 components of the coatings, fed into LPA, are added with thinner lines.

Figure 11. FWHMs of RCs [$^\circ$] recorded on wurtzite (002) peaks of Al-O-N films containing up to 8.7% O.

Figure 12. PFs of an Al-O-N film with 5% O. PF_{002} (a), top), recorded at 2θ=36.21, PF_{103} (b), middle) recorded at 2θ=66.12, and PF_{101} (c), bottom), recorded at 2θ=61.57. Plane inclination angles with respect to the film surface are designated as τ .

Figure 13. XPS spectra including fitted components of the photo e^- lines Al *2p* (top left), Si *2p* (top right), O *1s* (bottom left) and N *1s* (bottom right) of an Al-Si-O-N film containing 38.1% Al, 5.7% Si, 11.4% O and 44.9% N (at%).

Information Classification: General

Figure 14. Ψ and Δ with increasing wavelength, recorded for an Al-O-N coating with 34.6% O at the three reflection angles 50°, 60° and 70°, plotted with colored lines. The Cauchy simulation fitting the measurement is shown with a black dotted line.

Figure 15. GaFIB-SEM cross section through a crack propagating in line of sight which corresponds to the Si(100) plane. The Al-O-N film on the Si(100) substrate is covered by a Pt layer used for the GaFIB cut.

Figure 16. Diffractograms of Al-Si-O-N films derived from symmetric θ-2θ scans with Cu Kα. Normalized diffractgrams of samples with O contents of 0.1-12.6% and Si contents of 5.6-12.7% are shown in the 2θ range 30-40°. Diffractograms are distinguished into type I (blue), type II (green), type III (magenta) and type IV (purple). The positions of the Kα_1 wurtzite AlN reference peaks [43] at 33.26 for (100), at 36.07 for (002) and at 37.98 for (101) are shown in black and gray.

Figure 17. Residual stress, σ, in Al-Si-O-N coatings plotted versus the O content. σ was determined from films on extra-thin (30 μm Si(100) / 145 μm glass) substrates, as these bend instead of leading to stress relaxation cracks in the coatings.

Information Classification: General

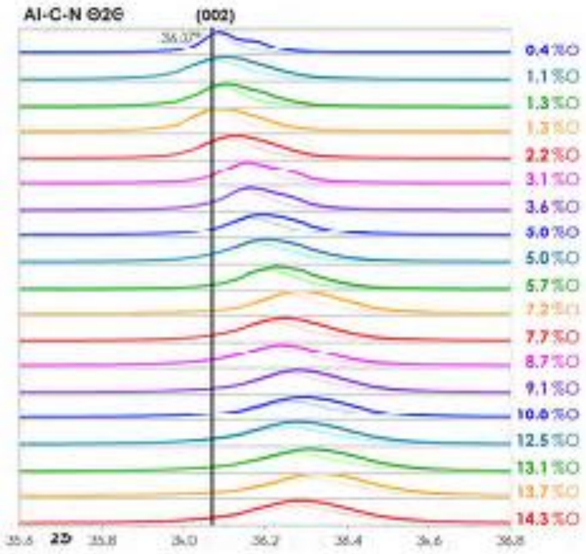

Figure 9. Normalized diffractogram line profiles from symmetric θ-2θ scans recorded with Cu Kα on Al-O-N thin films containing 0.4-14.3% O. The relevant 2θ range 35.6-36.8°, containing all signals arising from the coatings, is shown. The reference position of the wurtzite Cu Kα_1 peak [43] at 36.07 ° for (002) is shown in black. Fitted Cu Kα_1 components of the coatings, fed into LPA, are added with thinner lines.

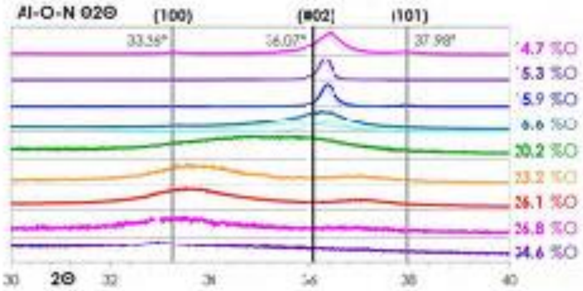

Figure 10. Normalized diffractogram line profiles from symmetric θ-2θ scans recorded with Cu Kα on Al-O-N thin films containing 14.7-34.6% O. The relevant 2θ range 30-40°, containing all signals arising from the coatings, is shown. Reference positions of wurtzite Cu Kα_1 peaks [43] at 33.26 for (100), 36.07° for (002) and 37.98 for (101) are shown in black and gray. Fitted Cu Kα_1 components of the coatings, fed into LPA, are added with thinner lines.

Information Classification: General

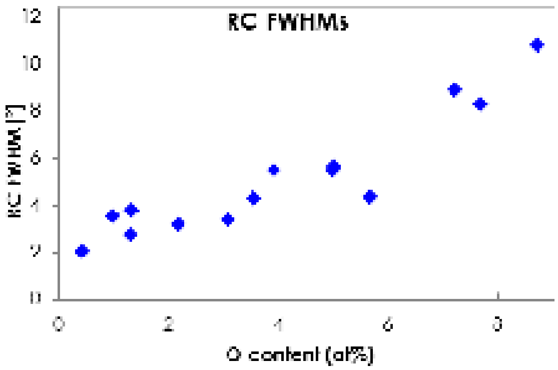

Figure 11. FWHMs of RCs [°] recorded on wurtzite (002) peaks of Al-O-N films containing up to 8.7% O.

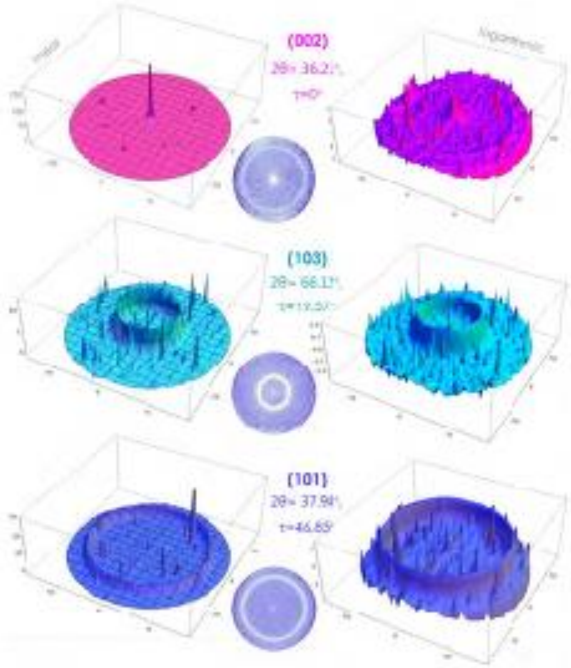

Figure 12. PFs of an Al-O-N film with 5% O. PF_{002} (a), top), recorded at 2θ–36.21, PF_{103} (b), middle) recorded at 2θ–66.12, and PF_{101} (c), bottom), recorded at 2θ–61.57. Plane inclination angles with respect to the film surface are designated as τ.

Information Classification: General

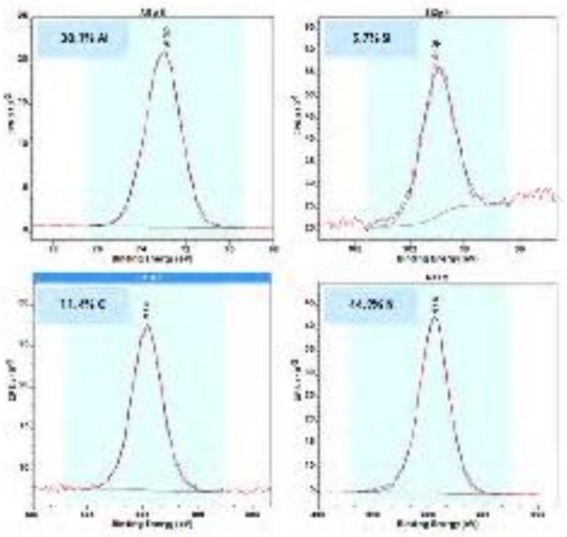

Figure 13. XPS spectra including fitted components of the photo e^{-} lines Al *2p* (top left), Si *2p* (top right), O *1s* (bottom left) and N *1s* (bottom right) of an Al-Si-O-N film containing 38.1% Al, 5.7% Si, 11.4% O and 44.9% N (at%).

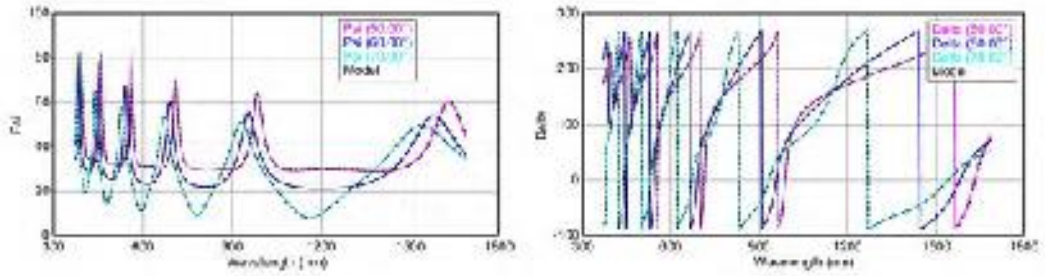

Figure 14. Ψ and Δ with increasing wavelength, recorded for an Al-O-N coating with 34.6% O at the three reflection angles 50°, 60° and 70°, plotted with colored lines. The Cauchy simulation fitting the measurement is shown with a black dotted line.

Information Classification: General

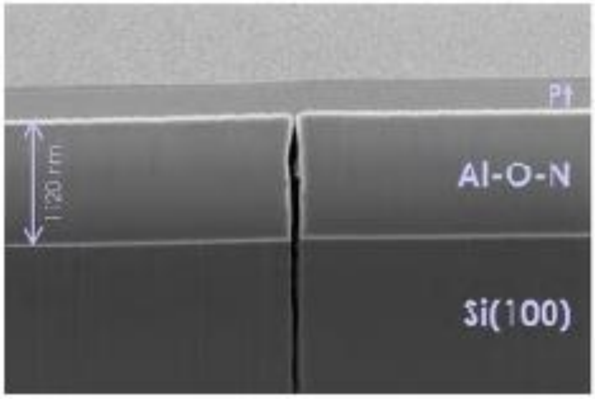

Figure 15. GaFIB-SEM cross section through a crack propagating in line of sight which corresponds to the Si(100) plane. The Al-O-N film on the Si(100) substrate is covered by a Pt layer used for the GaFIB cut.

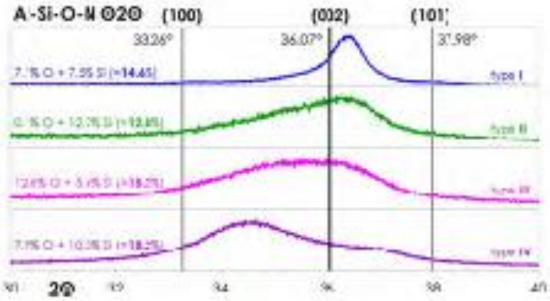

Figure 16. Diffractograms of Al-Si-O-N films derived from symmetric θ-2θ scans with Cu $K\alpha$. Normalized diffractgrams of samples with O contents of 0.1-12.6% and Si contents of 5.6-12.7% are shown in the 2θ range 30-40°. Diffractograms are distinguished into type I (blue), type II (green), type III (magenta) and type IV (purple). The positions of the $K\alpha_1$ wurtzite AlN reference peaks [43] at 33.26 for (100), at 36.07 for (002) and at 37.98 for (101) are shown in black and gray.

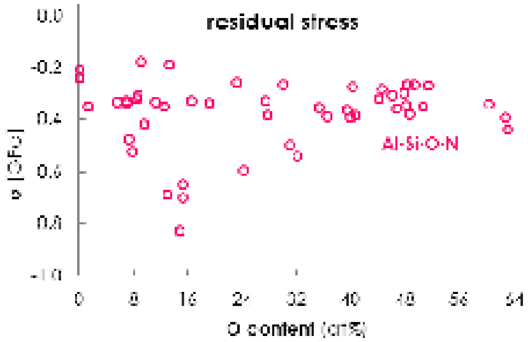

Figure 17. Residual stress, σ, in Al-Si-O-N coatings plotted versus the O content. σ was determined from films on extra-thin (30 μm Si(100) / 145 μm glass) substrates, as these bend instead of leading to stress relaxation cracks in the coatings.

Information Classification: General
